# Supplementary material for: HIVprotI: an integrated web based platform for prediction and design of HIV proteins inhibitors
Source: J Cheminform. 2018 Mar 9;10:12. doi: 10.1186/s13321-018-0266-y (PMC5845081; doi:10.1186/s13321-018-0266-y)
Supplement: Supplementary file 2 — Additional file 2. Source code of HIVProtI web server. [file 13321_2018_266_MOESM2_ESM.zip › HIVprotI_Source-code/hivprotI/weka-3-6-6/doc/weka/associations/FPGrowth.html]

FPGrowth


---


|  |  |  |
| --- | --- | --- |
| **Overview** \| **Package** \| **Class** \| **Tree** \| **Deprecated** \| **Index** \| **Help** \| **Weka's home** | |  |
| **PREV CLASS**   **NEXT CLASS** | **FRAMES**    **NO FRAMES**     **All Classes** |  |
| SUMMARY: NESTED \| FIELD \| CONSTR \| METHOD | DETAIL: FIELD \| CONSTR \| METHOD |  |


---


## weka.associations Class FPGrowth

```
java.lang.Object
  weka.associations.AbstractAssociator
      weka.associations.FPGrowth
```

**All Implemented Interfaces:**: java.io.Serializable, java.lang.Cloneable, Associator, CapabilitiesHandler, OptionHandler, RevisionHandler, TechnicalInformationHandler

---

``` public class FPGrowth extends AbstractAssociator implements OptionHandler, TechnicalInformationHandler ```

Class implementing the FP-growth algorithm for finding large item sets without candidate generation. Iteratively reduces the minimum support until it finds the required number of rules with the given minimum metric. For more information see:
